# Juvenile social experience generates differences in behavioral variation but not averages

**DOI:** 10.1093/beheco/ary185

**Published:** 2018-12-21

**Authors:** Nicholas DiRienzo, J Chadwick Johnson, Anna Dornhaus

**Affiliations:** 1 Department of Ecology and Evolutionary Biology, University of Arizona, Tucson, AZ, USA; 2 School of Mathematical and Natural Sciences, Arizona State University, Glendale, AZ, USA

**Keywords:** animal personality, black widow spider, developmental plasticity, individual variation, web structure

## Abstract

Developmental plasticity is known to influence the mean behavioral phenotype of a population. Yet, studies on how developmental plasticity shapes patterns of variation within populations are comparatively rare and often focus on a subset of developmental cues (e.g., nutrition). One potentially important but understudied developmental experience is social experience, as it is explicitly hypothesized to increase variation among individuals as a way to promote “social niches.” To test this, we exposed juvenile black widow spiders (*Latrodectus hesperus*) to the silk of conspecifics by transplanting them onto conspecific webs for 48 h once a week until adulthood. We also utilized an untouched control group as well as a disturbed group. This latter group was removed from their web at the same time points as the social treatment, but was immediately placed back on their own web. After repeatedly measuring adult behavior and web structure, we found that social rearing drove higher or significant levels of repeatability relative to the other treatments. Repeatability in the social treatment also decreased in some traits, paralleling the decreases observed in the disturbed treatments. Thus, repeated juvenile disturbance may decrease among-individual differences in adult spiders. Yet, social rearing appeared to override the effect of disturbance in some traits, suggesting a prioritization effect. The resulting individual differences were maintained over at least one-third of the adult lifespan and thus appear to represent stable, canalized developmental effects and not temporal state differences. These results provide proximate insight into how a broader range of developmental experiences shape trait variation.

## Teaser

Juvenile social experience increases adult behavioral differences, in some, but not all traits. This result indicates that juvenile experience may play an important role in shaping individual social niches. Unexpectedly, the disturbance from removing spiders from their containers generally reduced variation but increased how body size influenced behavior, suggesting that individuals have unique responses to different developmental experiences. These differences persisted for the duration of the experiment highlighting the long-term effects of developmental experience.

## INTRODUCTION

Within populations, among-individual variation in behavior is both ubiquitous and important for evolution and ecology (Chapman et al. 2011; Sih et al. 2012; Wolf and Weissing 2012). For example, in mosquitofish (*Gambusia affinis*), some individuals are consistently less social than others, and these less social individuals disperse farther when given the opportunity (Cote et al. 2010). Although these patterns of variation have been shown to be stable across significant periods of the adult lifespan in various organisms (Guenther et al. 2014; Fisher et al. 2015; Wuerz and Krüger 2015; DiRienzo and Montiglio 2016a), this variation among individuals, as well as average tendencies of the population, is also sensitive to the conditions during ontogeny (Edenbrow and Croft 2013; Careau et al. 2014; DiRienzo et al. 2015; Urszán et al. 2015; DiRienzo and Montiglio 2016b; Han and Dingemanse 2017; Horváth et al. 2017a). Yet, much of the research on the development of individual differences has focused on nutritional stress, with only a few studies considering a broader range of relevant ecological conditions. Other cues, specifically social cues that relate to population density, may be highly relevant as the value of a given strategy can depend on the individuals you interact with (Pruitt and Riechert 2011; Montiglio et al. 2017). Given the significant consequences of trait variation, it is critical to assess how a wider range of developmental cues affect the generation of that variation.

Developmental plasticity has long been studied in behavioral ecology, and recently researchers have begun applying the theory of adaptive developmental plasticity to an individual variation perspective. Of course, average phenotypes within a population are well known to respond to ontogenetic cues, which in theory allow an organism to “match” its phenotype to the predicted environment (West-Eberhard 2003). For example, field crickets are less aggressive when reared in high densities or with cues of high density, presumably to mitigate the cost of frequent agonistic interaction (Iba et al. 1995; DiRienzo et al. 2012). Yet, differences in several factors at the individual level could drive developmentally induced increases or decreases in variation around that mean population tendency. First, adult individuals are frequently limited in plasticity, often showing stable among-individual variation across time, conditions, and state (Sih et al. 2004; Schuett and Dall 2009; Westneat et al. 2011; Dammhahn and Almeling 2012; DiRienzo and Montiglio 2016b; DiRienzo and Aonuma 2017, 2018). Such limited adult plasticity should select for juveniles to “match” their phenotype to the predicted environment through developmental plasticity. Second, differences in genetics or early state may drive individuals to respond differently to the same cues (Stamps and Groothuis 2010; Snell-Rood 2013). Together, this could drive adaptive developmental responses that alter the amount of variation within a population. For example, nutritional stress tends to increase aggression in a population on average, but also amplifies among-individual variation in multiple species including widow spiders, rock lizards, and crickets (DiRienzo and Montiglio 2016b; Royauté and Dochtermann 2016; Horváth et al. 2017a). Such increases in variation could arise if initially small individuals do not increase aggression as much as larger individuals if there are costs to being a small and aggressive adult. Although research into developmentally driven individual differences has experienced an upswing both in theory and empirical research (Stamps and Groothuis 2010; Sweeney et al. 2013; DiRienzo et al. 2015; Favati et al. 2015; Liedtke et al. 2015; Urszán et al. 2015; DiRienzo and Montiglio 2016b; Han and Dingemanse 2017; Horváth et al. 2017a), much of the latter category has focused on how nutritional stress, either in quantity or macronutrient content, affects variation, with relatively few studies addressing how other experiences shape variation. Overall, a broader range of cues need to be studied if we are to gain a general understanding of how individual differences arise in response to the various relevant ecological conditions.

Juvenile social experience is one such important development cue as both nonsocial and social animals can have social interactions in their natal environment. Variation in early social experience, driven by differences in population density or cues of density, may result in different individual and population-level responses (Fenderson et al. 1968; Iba et al. 1995; Niemelä et al. 2012). From an average perspective, high densities generally reduce aggression towards conspecifics, possibly due to the high cost of fighting (Iba et al. 1995; Knell 2009; DiRienzo et al. 2012), yet relatively little is known how among-individual variation is affected. The social niche hypothesis suggests that repeated social interactions, as would occur in high densities, should drive individuals to occupy different behavioral niches as a way to reduce competitive interactions (Bergmüller and Taborsky 2010). In theory, such a response should increase among-individual variation, although recent studies on the topic have seen mixed results. From a nonontogenological perspective, several studies in social spiders have demonstrated that repeated interactions among artificially created colonies increase among-individual differences (Laskowski and Pruitt 2014; Modlmeier et al. 2014), suggesting that social stratification will naturally occur. Yet, evidence is mixed regarding the role of social experience during development in shaping individual differences. Bierbach et al. (2017) showed that in genetically identical clonal Amazon mollies (*Poecilia formosa*), among-individual differences in activity developed even when reared in identical conditions, and that the variation did not increase in response to social rearing (Bierbach et al. 2017). Yet, results from Urszán et al. (2015) are somewhat in contrast to this, as they found that socially reared *Rana dalmatina* tadpoles showed significant repeatability in activity and risk taking when reared in conjunction with predator cues, but not when reared in groups without predator cues (Urszán et al. 2015). These results provide differing degrees of support for the role of social experience in shaping population patterns of variation, highlighting the need to study such a question in a wider range of taxa and traits.

Black widow spiders (*Latrodectus spp.*) are an ideal model to study how development shapes behavior. Previous studies have shown that adult patterns of behavior and web structure are sensitive to developmental conditions. Specifically, food-stressed spiders are more aggressive and build webs with more gumfooted lines that aid in prey capture, whereas nonstressed spiders are less aggressive and build denser webs with more nonforaging structural lines that likely increase protection (DiRienzo and Montiglio 2016b). Repeatability of behavior across the adult lifespan is similarly affected, with food-stressed spiders showing greater levels of among-individual variation across a wider range of traits than their nonstressed peers (DiRienzo and Montiglio 2016b). Widow spiders also show state dependence in response to body condition such that reductions in mass drive increased aggression and building of gumfooted lines (Blackledge and Zevenbergen 2007; Zevenbergen et al. 2008; DiRienzo and Montiglio 2016b), although individual differences still remain indicating the stability of these traits and the potential long-term developmental effects. Furthermore, although historically they are asocial and aggressive towards conspecifics, they often can be found living in dense aggregations within both urban and natural environments (Salomon et al. 2010; Johnson et al. 2012; Trubl et al. 2012), suggesting that developmental plasticity through experiencing conspecific cues during ontogeny, or adult plasticity through repeated interaction (e.g. Laskowski and Pruitt 2014; Modlmeier et al. 2014), may drive behavioral changes that increase conspecific tolerance. Being reared in such high densities may increase trait variation through social niche specialization, which in turn reduces competition (Bergmüller and Taborsky 2010).

Here we investigate how juvenile social experience affects among-individual individual differences, measured as repeatability, in adult behavior and web structure of black widow spiders. We accomplish this by rearing juvenile spiders under 3 different conditions: social, where spiders experience another spider’s silk and associated pheromones for 48 h each week throughout development; disturbed, where spiders are removed from their containers twice a week and have their webs disturbed in the process; and control, which are not manipulated. The disturbed treatment allows us to account for the influence of web disturbance that happens in the social treatment when individuals are removed from their webs. We expect that, according to theory, social experience will increase consistent variation among individuals, but have little effect on the average phenotype of the population relative to control groups.

## MATERIALS AND METHODS

### Experimental design

Juvenile spiders at the third to fourth instar were collected throughout Davis, California in August, 2016. Spiders were brought into the laboratory at the University of Arizona in Tucson, Arizona, where each was given an individual plastic container (6 cm high × 8.5 cm diameter). Spiders were then randomly assigned to 1 of 3 rearing treatments: social, disturbed, or control. In the social treatment, one spider would be removed from its home container on a Tuesday and placed in the container of a second individual from the same treatment group, whereas that second spider was removed and placed in the container of the first. On Thursday, the social spiders were returned to their home containers. Spiders were transferred to a different conspecific’s container each week. Thus, each individual from the social treatment experienced the silk and associated pheromones of a new conspecific (but not direct contact to avoid cannibalism) for 48 h each week, and for the other 5 days in their own container if the temporary resident performed any web building. In the case where there was an odd number of spiders in the social treatment, 3 way swaps were performed (e.g., spider 1 to spider 2’s web, 2 to 3’s web, and 3 to 1’s web). Disturbed spiders were simply removed from their containers and immediately replaced on both Tuesday and Thursday. As a result, social spiders experienced both disturbance from removal as well as the conspecific cues. In both social and disturbed treatments, the spiders did not have to build a full new web as we left the remaining silk/web in their container after removal. Control spiders were allowed to mature untouched. We were unable to have a control group that was removed from their web without destroying it as removing the spider from their small container virtually always destroyed the web. All manipulations ended once an individual matured. All spiders were provided with 2 approximately body-sized *Acheta domesticus* crickets a week. A total of 105 spiders, 35 per treatment group began the experiment, with 28 control, 24 social rearing, and 23 disturbed spiders surviving to adulthood. The uneven size of the resulting treatment groups was due to either mortality, or in one case, a spider never maturing.

Once mature, cohorts were created of all spiders that matured within that month. This resulted in 3 total cohorts and allowed us to synchronize the testing within and across months. We assessed behavior and web structure once a month for 3 months using standardized methods (see below). In brief, each month we allowed individuals to build a web for 7 days inside a skeletionized cardboard box, after which we assessed web structure. Once assessed, we measured aggression 3 times within a day, 3 days in a row on each web. Thus, all spiders built 3 webs and had their behavior assayed 27 times, assuming that they did not build a web outside of the box which prevented either assay from being applied. All spiders were weighed prior to entering the web building structure.

### Web structure assays

**Figure 1:**
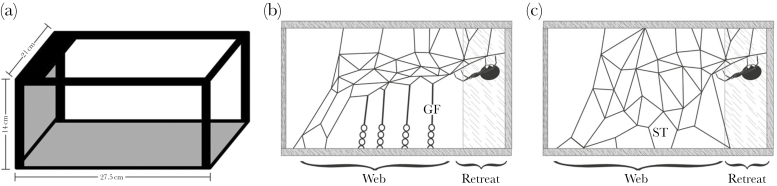
Diagram of the standardized web building structure (a). (b) and (c) illustrate side views of webs that contain either gumfooted lines (GF) or structural lines (ST) connecting to the ground. (b) and (c) Courtesy of Todd Blackledge.

We assessed individual web structure by allowing the spider to build for 7 days on a standardized structure. We created the structure by removing 3 walls from a cardboard box (L 27.5 × W 21 × H 14 cm), leaving only a 1-cm border of the box (Figure 1a). We also removed all but 3 cm of the top of the box, leaving a refuge for the spider connecting to the remaining rear wall. We covered the bottom and rear wall of the box in black paper to aid in the counting of the different line types. One structure was placed inside a larger plastic container (L 42.5 × W 27.75 × H 16.25 cm), after which the spider was added and given 7 days to build. After 7 days, we counted the number of gumfooted lines (as indicated by the sticky glue covering the lower portion of the line, Figure 1b—courtesy of Todd Blackledge) and structural lines (as indicated by their lack of glue and firm rooting to the floor, Figure 1c—courtesy of Todd Blackledge). Gumfooted lines represent web components that aid in prey capture, as those who build more gumfooted lines capture more prey (Zevenbergen et al. 2008), whereas structure lines and overall 3-dimensional density are hypothesized to aid in spider protection (Blackledge et al. 2003). We also weighed each web after the behavioral assays were complete. We did this by winding the web onto a plastic rod and then weighing it on a microbalance. This method gives an overall measure of web investment and directly correlates with web density (*r* = 0.8; Blackledge and Zevenbergen 2007).

### Behavioral assays

After building a web for 7 days, we assessed aggressive and subsequent retreat behavior. We applied a vibratory cue that has been previously shown to elicit a prey capture response in widow spiders (Keiser and Pruitt 2014; DiRienzo and Montiglio 2016a) and correlates with actual live prey capture success (DiRienzo N, unpublished data). We used a vibratory stimulus that vibrated at 100 Hz for approximately 1 s, after which it slowed for a half second. Attached to the end of the vibratory stimulus was a zip tie which allowed us to apply the cue to a single silk strand while also minimizing damage to the web. All spiders had the cue presented 3 times in 3 specific locations: 2 cm from the retreat, 2 cm from the far corner of the retreat, or in the middle of the 2 points. Each location was tested only once per day. A list of all possible presentation sequences (e.g., near, medium, far; medium, far, near) was created. Spiders were tested in a random order, but the presentation order was assigned sequentially going down the possible sequence list. Thus, the first randomly tested spider experienced presentation sequence A, the second spider sequence B, through sequence E, after which the seventh randomly tested spider received presentation sequence A again. This allowed for all presentation sequences to be applied an equal number of times over the duration of the experiment. The cue was presented 3 times for 15 s each time, with a 10-s break between applications. We noted if the spider attacked the prey cue, and if they did, if they retreated back to their shelter or remained out on their web. We conducted this assay 3 days in a row on each web.

### Statistical analysis

We assess if spiders matured at different rates using a multinomial logistic regression model. All spiders matured within 3 months, and thus, we predicted the likelihood of an individual maturing in any of the 3 bins as a byproduct of treatment. We fit the model in R version 3.4.3 using the package “nnet” version 7.3–12 (Ripley and Venables 2002; R Core Team 2015). We also assessed the effect of treatment on body mass using linear mixed models fit using the package “lme4” (Bates et al. 2015). We fit one model with treatment as a fixed effect and individual ID as a random effect, and another with just the random effect of ID. We then compared the models using AIC to determine the quality of fit (Akaike 1987). If the models differed in AIC by 2 or more, then the model with the lowest score was determined to be significantly better (Richards 2005).

To assess the effect of developmental treatment on behavior and web structure, we used generalized linear mixed models. Attack and retreat behavior were modeled separately, both using binomial error distributions. Fixed effects in the models included treatment, the number of the web they built (web number—a proxy for trial number), and distance the prey cue was presented (distance). We also included fixed effects of an interaction between body mass (scaled to a mean of zero and standard deviation of one) and treatment as our results indicated that treatment did have an effect on body mass. For web structure, we modeled the number of gumfooted lines and structure lines using Poisson errors, and web mass using Gaussian errors. Fixed effect structure was the same with the exception of not having the distance parameter. To assess if among-individual variance and repeatability estimates differed as a result of treatment, we fit all of the above models twice, but with differing random-effect structures. The “null” model contained a single random intercept for individual ID fit across all treatments, whereas the alternative model contained treatment-specific random effects. These latter models allows for one to estimate the variance and thus repeatability for each treatment group and assessment by their associated credible intervals. We also compared the Deviance Information Criteria between the models, as a lower DIC score indicates a better fit. Due to concerns of the short time intervals between behavioral assays within a day driving pseudorepeatability in attack and retreat behavior, we also ran an additional model for attack behavior that included trial number within the day as a random intercept. This model displayed essentially the same parameter estimates (Supplementary Table S1), and thus, the simpler model structure was used.

Models were fit using MCMCglmm (version 2.26) with 500 000 number of iterations, 50 000 burnin, and a thinning interval of 100 using R (Hadfield 2015). The quality of fit (mixing and convergence) was checked by visual inspection. All models were run 5 times to ensure the stability of results. Repeatability estimates calculated following the methods of Nakagawa and Schielzeth (2010) using the mean posterior variance estimates from the treatment-specific random intercept models (Nakagawa and Schielzeth 2010).

## RESULTS

### Treatment effects on maturation and body mass

Being in the social or disturbed treatment did not result in spiders maturing at a different rate from the control spiders (social: β = 0.906, SE = 0.618, *z* = 1.467, *P* = 0.142; disturbed: β = −0.022, SE = 0.571, *z* = −0.039, *P* = 0.969). Our mixed regression model did reveal that the model with treatment predicting body mass fit significantly better than the model without treatment (difference in AIC = 17.8). Parameter estimates of the model indicated that socially reared spiders maintained a lower body mass over the course of the experiment (β = −50.341, SE = 28.284), although the effect was marginal (*t* = −1.780, *P* = 0.075). Disturbed spiders showed no difference in body mass (β = 10.819, SE = 28.838, *t* = 0.375, *P* = 0.708).

### Treatment effects on mean behavioral tendencies

**Figure 2:**
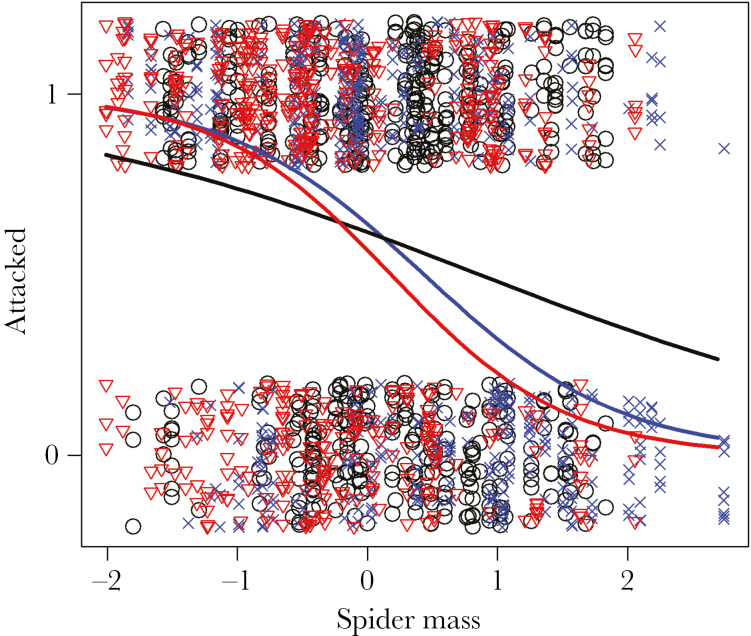
Figure depicting the treatment-specific differences of attack probablity in response to body mass (scaled to a mean of zero and standard deviation of one). Black line/circles = Control; red line/triangles = Social; blue line/crosses = Distrubed. Both social and disturbed treatment groups show significantly greater changes in attack behavior in response to body mass.

**Figure 3:**
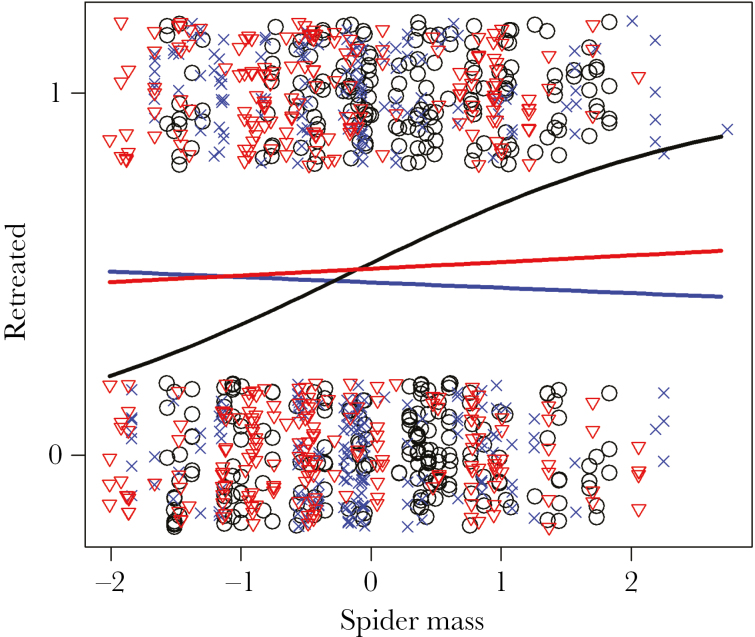
Figure depicting the treatment-specific differences of retreat probablity after an attack in response to body mass (scaled to a mean of zero and standard deviation of one). Black line/circles = Control; red line/triangles = Social; blue line/crosses = Distrubed. Both social and disturbed treatment groups show lower differences in retreat behavior in response to body mass.

Our results indicate that behavior was influenced by spider mass, although the magnitude of this effect differed by treatment. In terms of attack behavior, there was an overall negative effect of body mass (β = −0.569, 95% CI = −0.950: −0.139, *P* = 0.006) (Table 1), indicating that as spiders increased in body mass they became less likely to attack. Significant interaction terms suggest that this response is greater in magnitude in both social and disturbed treatments (social: β = −0.926, 95% CI = −1.654:−0.224, *P* = 0.008; disturbed: β = −0.761, 95% CI = −1.279: −0.162, *P* = 0.011) (Table 1; Figure 2). Retreat behavior showed a similar pattern whereby heavier spiders are more likely to retreat (β = 0.684, 95% CI = 0.175: 1.230, *P* = 0.008) (Table 2). Yet, a significant negative interaction in disturbed spiders (β = −0.751, 95% CI = −1.411: −0.132, *P* = 0.016), and trend in socially reared spiders (β = −0.597, 95% CI = −1.213: −0.064, *P* = 0.066) (Table 2) indicates that the retreat behavior in the treatment groups is not responsive to body mass (Figure 3). All of the web elements responded to body mass whereby heavier spiders built webs with fewer gumfooted lines (β = −0.831, 95% CI = −1.578: −0.074, *P* = 0.033) (Supplementary Table S2) but contained more structural lines (β = 0.442, 95% CI = 0.198: 0.675, *P* < 0.001) (Supplementary Table S3) and were heavier (β = 1.264, 95% CI = 0.011: 1.634, *P* < 0.001) (Supplementary Table S4), although there were no treatment-specific effects of body mass (Supplementary Tables S2–S4).

**Table 1:** Generalized linear mixed model output predicting the probability of attacking and retreating as a binary response

Random effects	Attack—Overall ID				Attack—Treatment-specific ID			
	Variance	L95% CI	U95% CI		Variance	L95% CI	U95% CI	
ID	5.656	3.373	8.325					
ID: Social					14.106	5.295	26.109	
ID: Disturbed					2.517	0.831	4.701	
ID: Control					5.137	1.892	9.147	
units	1	1	1		1.000	1.000	1.000	
Fixed effects	β	L95% CI	U95% CI	pMCMC	β	L95% CI	U95% CI	pMCMC
(Intercept)	2.944	1.893	4.019	0.000	2.936	1.912	4.016	0.000
Mass	−0.581	−1.019	−0.195	0.007	−0.569	−0.950	−0.139	0.006
Disturbed	0.094	−1.295	1.478	0.884	0.079	−1.053	1.269	0.887
Social	−0.225	−1.607	1.140	0.752	−0.298	−2.200	1.448	0.746
Distance	−1.018	−1.205	−0.826	0.000	−1.022	−1.212	−0.840	0.000
Web number	−0.206	−0.394	−0.001	0.040	−0.200	−0.394	−0.004	0.042
Mass * Disturbed	−0.815	−1.440	−0.272	0.012	−0.761	−1.279	−0.162	0.011
Mass * Social	−0.670	−1.338	−0.032	0.045	−0.926	−1.654	−0.224	0.008
DIC	1625.350				1621.575			

The control treatment group is set as the baseline. A total of 1800 attack observations are made over 75 individuals.

**Table 2:** Generalized linear mixed model output predicting the probability of attacking and retreating as a binary response

Random effects	Retreat—Overall ID				Retreat—Treatment-specific ID			
	Variance	L95% CI	U95% CI		Variance	L95% CI	U95% CI	
ID	1.729	0.846	2.799					
ID: Social					0.959	0.104	2.157	
ID: Disturbed					0.939	0.000	2.163	
ID: Control					4.518	1.342	8.903	
Units	1.000	1.000	1.000		1.000	1.000	1.000	
Fixed effects	β	L95% CI	U95% CI	pMCMC	β	L95% CI	U95% CI	pMCMC
(Intercept)	−0.293	−1.139	0.520	0.497	−0.233	−1.324	0.806	0.640
Mass	0.584	0.137	1.065	0.014	0.684	0.175	1.230	0.008
Disturbed	−0.152	−1.021	0.739	0.731	−0.253	−1.342	0.769	0.633
Social	0.049	−0.817	0.992	0.925	−0.095	−1.112	0.999	0.848
Distance	0.283	0.081	0.491	0.005	0.286	0.085	0.492	0.007
Web number	−0.094	−0.346	0.123	0.444	−0.094	−0.318	0.146	0.428
Mass * Disturbed	−0.666	−1.272	−0.024	0.040	−0.751	−1.411	−0.132	0.016
Mass * Social	−0.473	−1.121	0.143	0.131	−0.597	−1.213	0.064	0.066
DIC	1243.373				1241.718			

The control treatment group is set as the baseline. A total of 985 retreat observations are made over 75 individuals.

### Treatment effects on repeatability and among-individual variation

**Figure 4:**
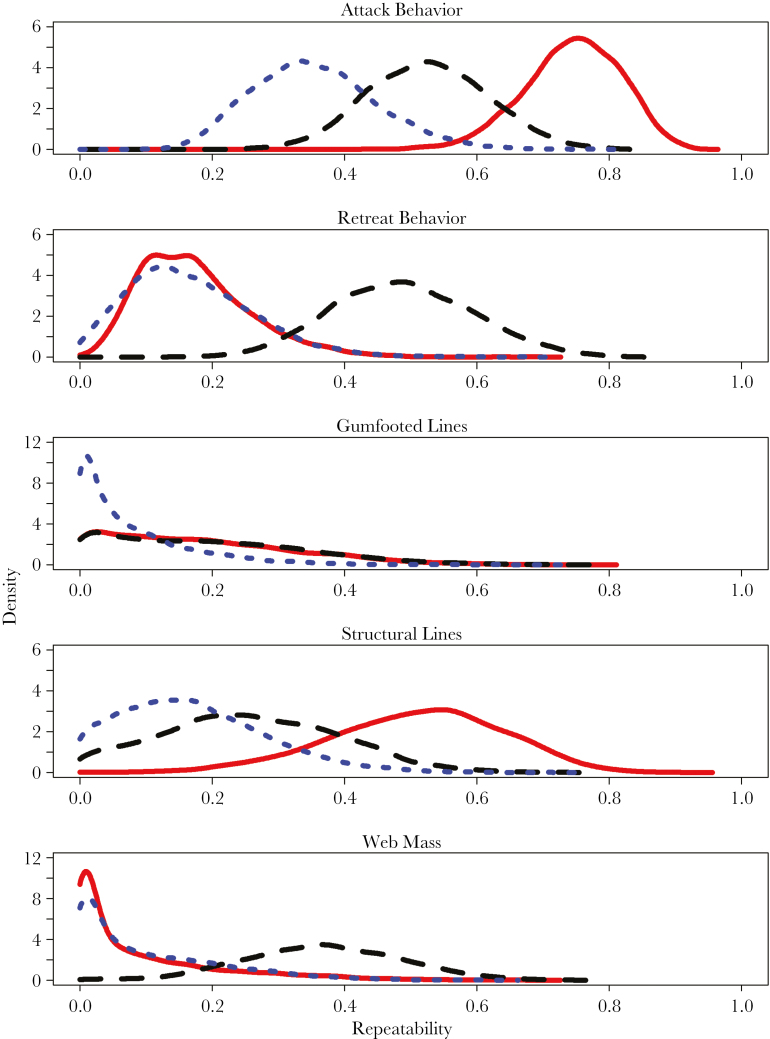
Posterior densities of repeatability estimates for the 3 treatment groups. Red solid line = social treatment; black long-dashed line = control treatment; blue short-dashed line = disturbed treatment.

We found mixed results in terms of treatment effects on repeatability and among-individual variation. Attack behavior showed the strongest effects of treatment, whereby social individuals had extremely high repeatability (*r* = 0.745), with disturbed spiders having comparatively low repeatability (*r* = 0.355) and control spiders falling at an intermediate level (*r* = 0.526) (Table 3; Figure 4). The DIC of the model fitting treatment-specific random intercepts was nearly 4 units lower than the single random intercept model suggesting that treatments do differ in repeatability and among-individual variance (Table 1). The treatment-specific model for retreat behavior also had a lower DIC (Table 2), although the patterns of variance differed from attack behavior. Specifically, control spiders displayed the highest repeatability (*r* = 0.488), with socially reared ones displaying lower, but significant repeatability (*r* = 0.173) as indicated by the credibility interval not abutting zero (95% CI = 0.034: 0.343) (Table 3). Disturbed spiders displayed low repeatability as well (*r* = 0.167), and the credibility interval did abut zero (95% CI = 0.000: 0.335) (Table 3). Variation in web structure demonstrated similarly variable results. Socially reared spiders showed significant repeatability in structural lines (*r* = 0.509, 95% CI = 0.244: 0.341), whereas the disturbed and control treatments did not (disturbed *r* = 0.170, 95% CI = 0.000: 0.315; control *r* = 0.257, 95% CI = 0.000: 0.474) (Table 3). Neither treatment group showed repeatability in the number of gumfooted lines or web mass, although the web mass of control spiders was repeatable (*r* = 0.367, 95% CI = 0.146: 0.599) (Table 3).

**Table 3:** Repeatability (R) on the link scale and among-individual variance (AIvar) estimates along with their 95% credibility intervals (R CI and AIvar CI, respectively)

Trait	Treatment	R	R CI	AIvar	AIvar CI
Attack	Control	0.526	0.360: 0.710	5.137	1.892: 9.147
	Disturbed	0.355	0.175: 0.531	2.517	0.831: 4.701
	Social	0.745	0.600: 0.876	14.106	5.295: 26.109
Retreat	Control	0.488	0.291: 0.702	4.518	1.342: 8.903
	Disturbed	0.167	0.000: 0.335	0.939	0.000: 2.163
	Social	0.173	0.034: 0.343	0.959	0.104: 2.157
Gum	Control	0.189	0.000: 0.450	1.349	0.000: 3.881
	Disturbed	0.076	0.000: 0.252	0.564	0.000: 2.025
	Social	0.180	0.000: 0.437	1.263	0.000: 3.644
Structural	Control	0.257	0.000: 0.474	0.216	0.000: 0.488
	Disturbed	0.170	0.000: 0.370	0.226	0.000: 0.574
	Social	0.509	0.244: 0.744	0.671	0.132: 1.378
Web mass	Control	0.367	0.146: 0.599	0.798	0.148: 1.610
	Disturbed	0.100	0.000: 0.315	0.159	0.000: 0.552
	Social	0.090	0.000: 0.341	0.147	0.000: 0.604

All estimates are obtained from the posterior distributions of the fitted MCMC models.

## DISCUSSION

Our results indicate that juvenile social cues can increase among-individual variation in repeatability in some, but not all traits. We found that attack behavior and the number of structural lines showed higher or significant repeatability than the other treatments, whereas other traits in the social treatment showed nonsignificant repeatability when the control treatment showed significant repeatability. Often the disturbed treatment, which was intended to control for the effect of removal in the social treatment, actually displayed lower and nonsignificant repeatability compared with the control treatment, suggesting an effect of disturbance on trait variation. Finally, body mass–dependent treatment effects were found in both behavioral measures. These interactions resulted in heavier spiders being less likely to attack in the social treatments, and generally less likely to retreat as well.

Our results provide support that being reared with social cues can increase among-individual variation in behavior, but not in every trait. We found the largest effect of social rearing on attack behavior, which may be due to the general relevance of that trait when interacting with conspecifics. In a social context, aggression may be highly costly if an individual does not possess the body size or underlying physiology to support that behavior, and thus already less-aggressive juveniles may want to further decrease aggression if conspecific density is high (Knell 2009; DiRienzo et al. 2012). The number of structural lines built also showed significant repeatability when the other treatments did not. There is evidence to suggest that spiders with more structural lines are better able to defend their webs against conspecifics (DiRienzo N, unpublished data), and thus, different social niches (e.g., aggressive vs. nonaggressive spiders) may also utilize different web-building strategies to further solidify their niche. Although aggression and structural lines responded to social rearing, the other measured traits did not. One explanation is that these traits are simply less relevant during social interactions. Gumfooted lines are used specifically for foraging, for example, and may have little impact on conspecific interactions. Generally considering the relevance of a specific behavioral trait to social interactions could inform why individuals do not always respond to social cues. For example, Bierbach et al. (2017) showed no effect of social rearing on the activity levels in Amazon Mollies, but that could be a result of swimming distance, the measured trait, not being important for interactions with conspecifics. Alternatively, such differential outcomes could be a species-specific result that is dependent on the importance of social interactions in that system. Widow spiders are facultatively social (or at least tolerant of conspecifics) and can be found living in high densities with extensive conspecific contact (Salomon et al. 2010), or in extremely low densities in desert environments. Being flexible in response to conspecific density may be useful when social conditions can be so variable. The specific spiders used in this study were collected from high-density urban populations, which may further predispose them to displaying such responsiveness to developmental cues. In general, whether a species responds to social rearing is likely contingent on the natural history of the system and the relevance of the behavior being measured to social interactions.

An unexpected outcome of this study was the persistent negative effect of repeated juvenile disturbance on individual variation coupled with increased size-dependent behavior. One explanation for this pattern is that under such frequent disruption, persistent differences are less favored, and instead plasticity and/or stochasticity in behavior is favored (Biro and Adriaenssens 2013; Westneat et al. 2015). Such findings have implications for species responses to human-induced environmental changes and urbanization, as a key characteristic of those habitats is frequent and unpredictable disturbance (Ditchkoff et al. 2006; Sih et al. 2011; McDonnell and Hahs 2015). Indeed, urban environments have been hypothesized to select for increased plasticity and/or state dependence, and research as often supported this hypothesis (Carrete and Tella 2017; Cook et al. 2017; Kralj-Fišer et al. 2017; Hardman and Dalesman 2018). For example, urban populations of great tits (*Parus major)* are both more aggressive and show less among-individual variation than their rural counterparts (Hardman and Dalesman 2018). Our results provide additional evidence that disturbed environments promote the development of unique individual and population-level patterns of behavior, which are likely to have implications for a species to thrive, or not under anthropogenic change. Given widow spiders excel at both invading new and novel habitats as well as persisting in high densities in urban environments (Johnson et al. 2012; Trubl et al. 2012), future studies should consider the role of developmental plasticity in their adaptability.

Although developmental cues are often studied singly, most animals experience multiple cues concurrently and as a result may make decisions regarding which one to respond to. Our results indicate that in some traits juveniles appear to prioritize social cues over disturbance cues. This was evident in attack behavior and the number of structural lines built, as even the social treatment experienced disturbance as a byproduct of the manipulation, yet displayed generally higher or significant repeatability and among-individual variance estimates relative to the disturbed treatment, whereas the true control fell at an intermediate level. It is unknown if this prioritization effect occurs because there are greater fitness costs to not responding to social cues, or if another mechanism is at play such as varying frequency or duration of the cues. The relative duration, or consistency of the social cues (48 h), may simply give more accurate information to the juvenile (Gabriel et al. 2005; Leimar et al. 2006) and thus is more likely to express a developmentally plastic response. Yet, in nature, individuals are unlikely to be consistently disturbed, but social cues will consistently be present when population densities are high, so the above scenario is not unrealistic (Salomon et al. 2010). Such a prioritization may drive increased context-dependent fitness outcomes whereby the individual will have high fitness in social settings, but pay costs when disturbance is also common. Given the complex nature of these interactions, and that no animal develops with only a single developmental cue, future research should be directed at looking at how juvenile cue interactions affect development at multiple levels of variation.

Although we did not set out to test this question explicitly, a persistent question in the literature is how stable among-individual differences are over time. Yet, here we show developmentally driven differences in repeatability and variance that were measureable over the 4-month period after maturation, which is significantly relative to their typical 1-year lifespan (although longer has been observed in the lab; DiRienzo N, personal observation). Furthermore, these differences persisted after the cues were removed and were not simply a result of size or measured state differences, suggesting that these are developmentally canalized and possibly rooted in alterations in a less-flexible underlying physiological mechanism (Nishi et al. 2010; DiRienzo and Aonuma 2017). One fundamental question that arises is how important developmental experience is relative to adult experience (e.g., developmental vs. activational plasticity) as both are known to affect variation (development: results within; DiRienzo et al. 2015; Urszán et al. 2015; DiRienzo and Montiglio 2016b; activational: Bell and Sih 2007; Frost et al. 2007; Laskowski and Pruitt 2014; Modlmeier et al. 2014; Horváth et al. 2017a, 2017b). Presumably, experience early in development is more potent (Snell-Rood 2013), yet cues may vary during development which may reduce the effect (Mangel 1990). Similarly, individuals may experience activational cues repeatedly during adulthood (Bell and Sih 2007; Laskowski and Pruitt 2014), which may reinforce or generate individual differences. Although this study was not designed to test for the relative contribution of each, future studies should investigate how the magnitude and frequency of both experiences shape patterns of variation.

The differential responses of variation to the developmental cues also allow one to test the predictions made by adaptive vs. nonadaptive hypotheses for the existence of personality. Specifically if individual differences arose from noise or mutation, one would predict no influence of specific environments on the amount of variation among individuals (Bürger et al. 1989; Lynch et al. 1998; Santiago 1998; Dall et al. 2004; Sih et al. 2004; Verweij et al. 2012; Careau et al. 2014). Similarly, as our treatments increased developmental stress, one would predict an increase in both treatments if developmental noise drove individual differences (Hoffmann and Hercus 2000; Archer et al. 2003; Vogt et al. 2008; Kain et al. 2012; Freund et al. 2013; Stamps et al. 2013; Lazić et al. 2015). Yet, our results show clear increases and decreases in trait variation. These results follow predictions that arise from the adaptive developmental plasticity hypothesis, whereby an individual’s genotype and condition dictate the level and direction of plasticity expressed (West-Eberhard 2003; Stamps and Groothuis 2010; Stamps and Krishnan 2014), and thus, bidirectional responses are expected. It could be argued that developmental stressors could create nonadaptive decreases in variation if they cause all individuals to encounter the same physiological constraint. Although possible, it does not seem to be what drove the reduction in variation due to disturbance as the social treatment, which often showed high variation, was also disturbed as part of the treatment. Of course, it is likely that developmental plasticity does not operate in isolation, and that developmental or mutational noise may also contribute to variation (Archer et al. 2003; Bierbach et al. 2017). Yet, the general collapse of variation in the disturbed treatment suggests that even if developmental noise drives some level of individual differences (Sih et al. 2012; DiRienzo and Montiglio 2016a), they can be reduced through developmental plasticity. We did not measure any fitness proxies in this study, and thus, even though the observed responses fit the patterns expected by adaptive hypotheses, future studies should focus on measuring the actual fitness consequences of these developmental responses.

## FUNDING

This work was supported by a University of Arizona Postdoctoral Excellence in Research and Teaching Fellowship (NIH # 5K12GM000708-17) awarded to N.D. and by the National Science Foundation (grant no. IOS-1455983 to A.D.).

## Supplementary Material

Supplemetary File 1

Supplemetary File 2

Supplemetary File 3

Supplemetary File 4
