# Supplementary material for: Juvenile social experience generates differences in behavioral variation but not averages
Source: Behav Ecol. 2018 Dec 21;30(2):455–64. doi: 10.1093/beheco/ary185 (PMC6450201; doi:10.1093/beheco/ary185)
Supplement: Supplemetary File 1 [file ary185_suppl_supplemetary_file-1.docx]

|  | **Attack – Treatment Specific ID** | | | |
| --- | --- | --- | --- | --- |
| Random Effects | Variance | L95% CI | U95% CI |  |
| ID : Social | 12.174 | 4.538 | 23.004 |  |
| ID : Disturbed | 2.353 | 0.738 | 4.491 |  |
| ID : Control | 6.432 | 2.481 | 11.458 |  |
| Trial within day | 5.317 | 0.000 | 5.547 |  |
| units | 1.000 | 1.000 | 1.000 |  |
| Fixed Effects | ß | L95% CI | U95% CI | pMCMC |
| (Intercept) | 2.975 | 1.383 | 4.476 | 0.015 |
| Mass | -1.079 | -1.373 | -0.835 | 0.000 |
| Disturbed | -0.055 | -1.243 | 1.162 | 0.920 |
| Social | -0.230 | -2.006 | 1.499 | 0.793 |
| Distance | -1.016 | -1.196 | -0.831 | 0.000 |
| Web number | -0.185 | -0.386 | -0.001 | 0.062 |
| Mass * Disturbed | 2.975 | 1.383 | 4.476 | 0.015 |
| Mass * Social | -1.079 | -1.373 | -0.835 | 0.000 |
| DIC | 1619.689 |  |  |  |
|  |  |  |  |  |
|  |  |  |  |  |

Table S1: Generalized linear mixed model output predicting the probability of attacking and retreating as a binary response. This model included an additional random effect to ensure that pseudo repeatability did not drive the increased variance. The functionally similar treatment variances suggests that this is not an issue, and thus simpler models are presented in the main text. The control treatment group was set as the baseline. A total of 1800 attack observations were made over 76 individuals.

|  | **Gumfooted – Overall ID** | | | | **Gumfooted – Treatment Specific ID** | | | |
| --- | --- | --- | --- | --- | --- | --- | --- | --- |
| Random Effects | Variance | L95% CI | U95% CI |  | Variance | L95% CI | U95% CI |  |
| ID | 0.645 | 0.000 | 1.668 |  |  |  |  |  |
| ID : Social |  |  |  |  | 1.263 | 0.000 | 3.644 |  |
| ID : Disturbed |  |  |  |  | 0.564 | 0.000 | 2.025 |  |
| ID : Control |  |  |  |  | 1.349 | 0.000 | 3.881 |  |
| units | 4.961 | 3.299 | 6.829 |  | 4.892 | 3.233 | 6.607 |  |
| Fixed Effects | ß | L95% CI | U95% CI | pMCMC | ß | L95% CI | U95% CI | pMCMC |
| (Intercept) | 2.035 | 0.977 | 3.162 | 0.000 | 2.025 | 0.890 | 3.245 | 0.003 |
| Mass | -0.861 | -1.593 | -0.162 | 0.015 | -0.831 | -1.578 | -0.074 | 0.033 |
| Disturbed | -0.021 | -0.980 | 0.963 | 0.959 | 0.030 | -0.965 | 1.015 | 0.958 |
| Social | -0.274 | -1.241 | 0.715 | 0.558 | -0.276 | -1.369 | 0.910 | 0.615 |
| Web number | -0.518 | -0.961 | -0.049 | 0.023 | -0.531 | -0.994 | -0.111 | 0.019 |
| Mass * Disturbed | 0.383 | -0.581 | 1.314 | 0.420 | 0.370 | -0.532 | 1.393 | 0.436 |
| Mass * Social | 0.067 | -0.931 | 1.034 | 0.875 | -0.002 | -0.980 | 1.109 | 0.997 |
| DIC | 899.741 |  |  |  | 898.871 |  |  |  |
|  |  |  |  |  |  |  |  |  |
|  |  |  |  |  |  |  |  |  |

Table S2: Generalized linear mixed model output predicting the number of gumfooted lines built. The control treatment group was set as the baseline. A total of 195 webs were built by 75 individuals over the course of the experiment.

|  | **Structural – Overall ID** | | | | **Structural – Treatment Specific ID** | | | |
| --- | --- | --- | --- | --- | --- | --- | --- | --- |
| Random Effects | Variance | L95% CI | U95% CI |  | Variance | L95% CI | U95% CI |  |
| ID | 0.300 | 0.105 | 0.505 |  |  |  |  |  |
| ID : Social |  |  |  |  | 0.671 | 0.132 | 1.378 |  |
| ID : Disturbed |  |  |  |  | 0.226 | 0.000 | 0.574 |  |
| ID : Control |  |  |  |  | 0.216 | 0.000 | 0.488 |  |
| units | 0.494 | 0.353 | 0.664 |  | 0.505 | 0.356 | 0.672 |  |
| Fixed Effects | ß | L95% CI | U95% CI | pMCMC | ß | L95% CI | U95% CI | pMCMC |
| (Intercept) | 2.692 | 2.316 | 3.090 | 0.000 | 2.702 | 2.304 | 3.075 | 0.000 |
| Mass | 0.438 | 0.205 | 0.686 | 0.001 | 0.442 | 0.198 | 0.675 | 0.000 |
| Disturbed | -0.331 | -0.749 | 0.076 | 0.120 | -0.331 | -0.710 | 0.053 | 0.095 |
| Social | -0.272 | -0.663 | 0.122 | 0.197 | -0.317 | -0.793 | 0.153 | 0.183 |
| Web number | 0.097 | -0.048 | 0.230 | 0.166 | 0.095 | -0.055 | 0.233 | 0.204 |
| Mass * Disturbed | -0.109 | -0.445 | 0.224 | 0.502 | -0.089 | -0.412 | 0.228 | 0.577 |
| Mass * Social | 0.070 | -0.299 | 0.383 | 0.681 | 0.056 | -0.304 | 0.411 | 0.742 |
| DIC | 1273.047 |  |  |  | 1272.308 |  |  |  |
|  |  |  |  |  |  |  |  |  |
|  |  |  |  |  |  |  |  |  |

Table S3: Generalized linear mixed model output predicting the number of structural lines built. The control treatment group was set as the baseline. A total of 195 webs were built by 75 individuals over the course of the experiment.

|  | **Web Mass – Overall ID** | | | | **Web Mass – Treatment Specific ID** | | | |
| --- | --- | --- | --- | --- | --- | --- | --- | --- |
| Random Effects | Variance | L95% CI | U95% CI |  | Variance | L95% CI | U95% CI |  |
| ID | 0.466 | 0.108 | 0.850 |  |  |  |  |  |
| ID : Social |  |  |  |  | 0.147 | 0.000 | 0.604 |  |
| ID : Disturbed |  |  |  |  | 0.159 | 0.000 | 0.552 |  |
| ID : Control |  |  |  |  | 0.789 | 0.148 | 1.610 |  |
| units | 1.196 | 0.878 | 1.535 |  | 1.286 | 0.995 | 1.615 |  |
| Fixed Effects | ß | L95% CI | U95% CI | pMCMC | ß | L95% CI | U95% CI | pMCMC |
| (Intercept) | 3.593 | 3.016 | 4.088 | 0.000 | 3.601 | 3.028 | 4.203 | 0.000 |
| Mass | 1.247 | 0.917 | 1.587 | 0.000 | 1.263 | 0.911 | 1.634 | 0.000 |
| Disturbed | -0.226 | -0.811 | 0.289 | 0.408 | -0.212 | -0.713 | 0.330 | 0.441 |
| Social | -0.196 | -0.752 | 0.321 | 0.474 | -0.168 | -0.693 | 0.361 | 0.532 |
| Web number | -0.176 | -0.385 | 0.024 | 0.090 | -0.189 | -0.401 | 0.013 | 0.080 |
| Mass * Disturbed | -0.417 | -0.865 | 0.056 | 0.078 | -0.399 | -0.859 | 0.040 | 0.078 |
| Mass * Social | -0.369 | -0.840 | 0.127 | 0.130 | -0.309 | -0.796 | 0.151 | 0.196 |
| DIC | 634.492 |  |  |  | 639.467 |  |  |  |
|  |  |  |  |  |  |  |  |  |
|  |  |  |  |  |  |  |  |  |

Table S4: Linear mixed model output for the mass of the web built. The control treatment group was set as the baseline. A total of 195 webs were built by 75 individuals over the course of the experiment.
