# Supplementary material for: Juvenile social experience generates differences in behavioral variation but not averages
Source: Behav Ecol. 2018 Dec 21;30(2):455–64. doi: 10.1093/beheco/ary185 (PMC6450201; doi:10.1093/beheco/ary185)
Supplement: Supplemetary File 2 [file ary185_suppl_supplemetary_file-2.docx]

**Photo for web publication:** Female western black widow (*L. hesperus*) attacking a vibratory cue simulating live prey.


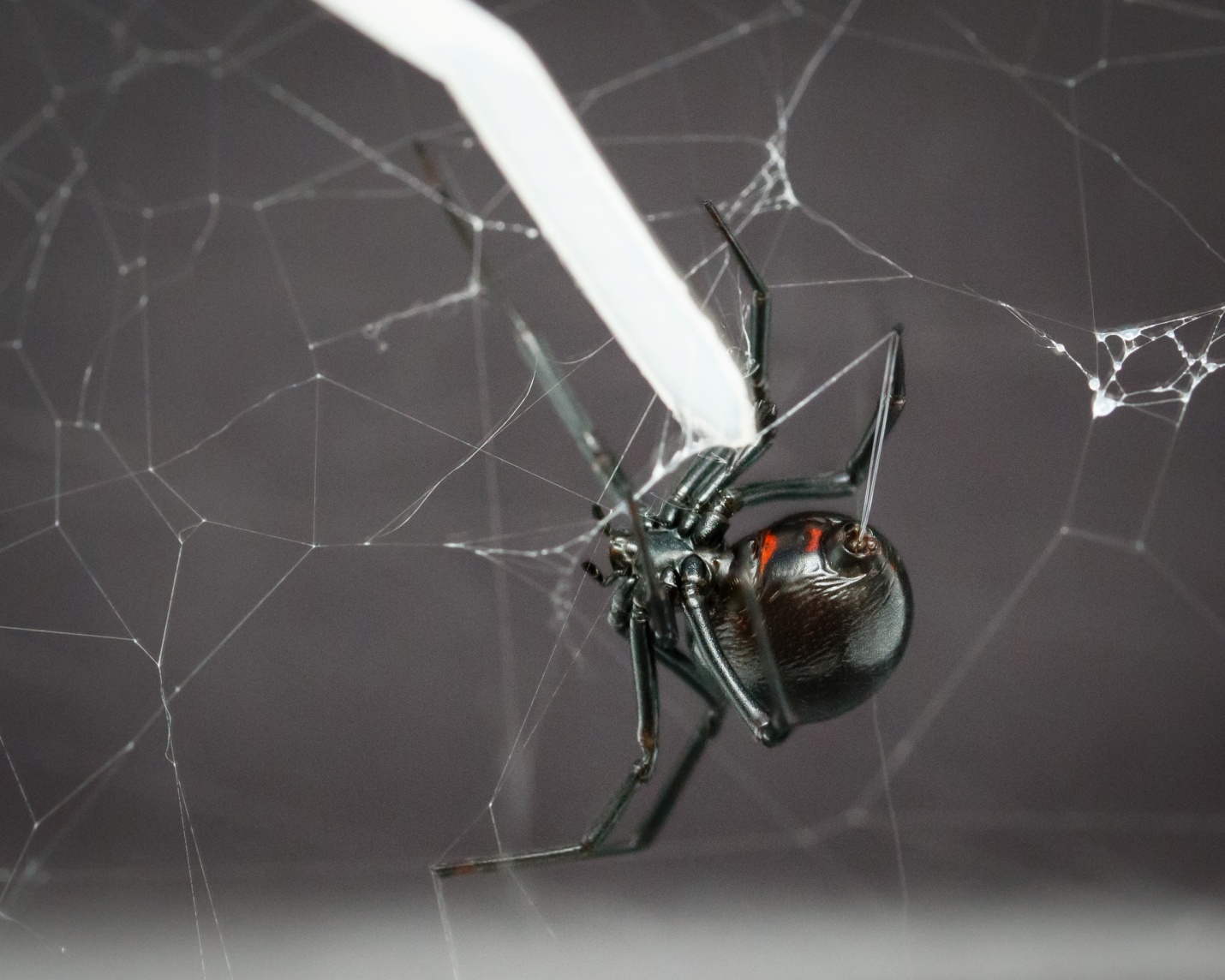


**Photo for web publication:** Female western black widow (*L. hesperus*).

**
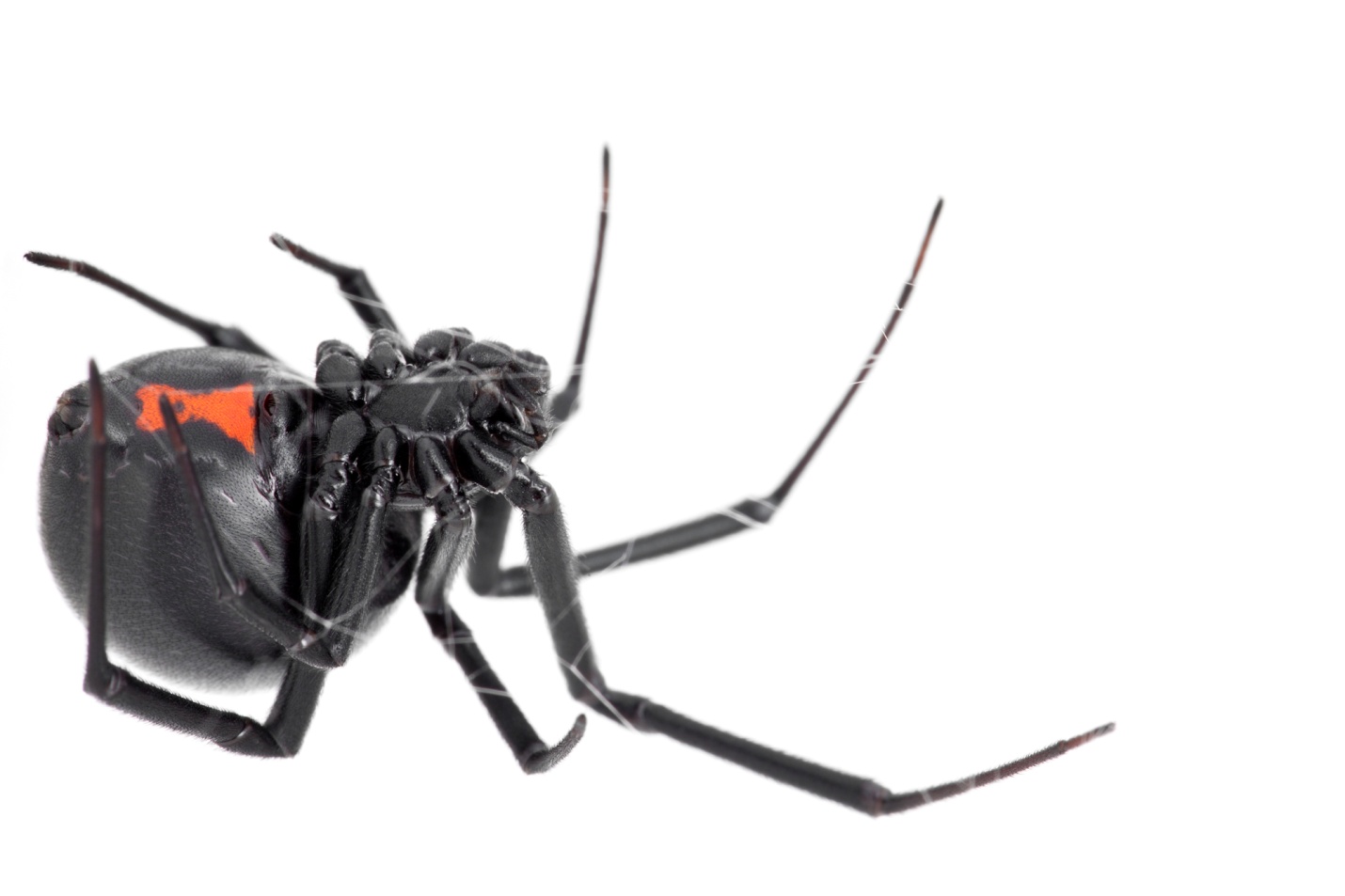
**
